# A Walk Through the Maze of Secondary Metabolism in Orchids: A Transcriptomic Approach

**DOI:** 10.3389/fpls.2022.837563

**Published:** 2022-04-29

**Authors:** Devina Ghai, Arshpreet Kaur, Parvinderdeep S. Kahlon, Sandip V. Pawar, Jaspreet K. Sembi

**Affiliations:** ^1^Department of Botany, Panjab University, Chandigarh, India; ^2^Chair of Phytopathology, TUM School of Life Sciences, Technical University of Munich, Freising, Germany; ^3^University Institute of Pharmaceutical Sciences, Panjab University, Chandigarh, India

**Keywords:** secondary metabolism, transcriptome, orchids, alkaloids, flavonoids

## Abstract

Orchids have a huge reservoir of secondary metabolites making these plants of immense therapeutic importance. Their potential as curatives has been realized since times immemorial and are extensively studied for their medicinal properties. Secondary metabolism is under stringent genetic control in plants and several molecular factors are involved in regulating the production of the metabolites. However, due to the complex molecular networks, a complete understanding of the specific molecular cues is lacking. High-throughput omics technologies have the potential to fill up this lacuna. The present study deals with comparative analysis of high-throughput transcript data involving gene identification, functional annotation, and differential expression in more than 30 orchid transcriptome data sets, with a focus to elucidate the role of various factors in alkaloid and flavonoid biosynthesis. Comprehensive analysis of the mevalonate (MVA) pathway, methyl-d-erythritol 4-phosphate (MEP) pathway, and phenylpropanoid pathway provide specific insights to the potential gene targets for drug discovery. It is envisaged that a positive stimulation of these pathways through regulation of pivotal genes and alteration of specific gene expression, could facilitate the production of secondary metabolites and enable efficient tapping of the therapeutic potential of orchids. This further would lay the foundation for developing strategies for genetic and epigenetic improvement of these plants for development of therapeutic products.

## Introduction

**Figure 1:**
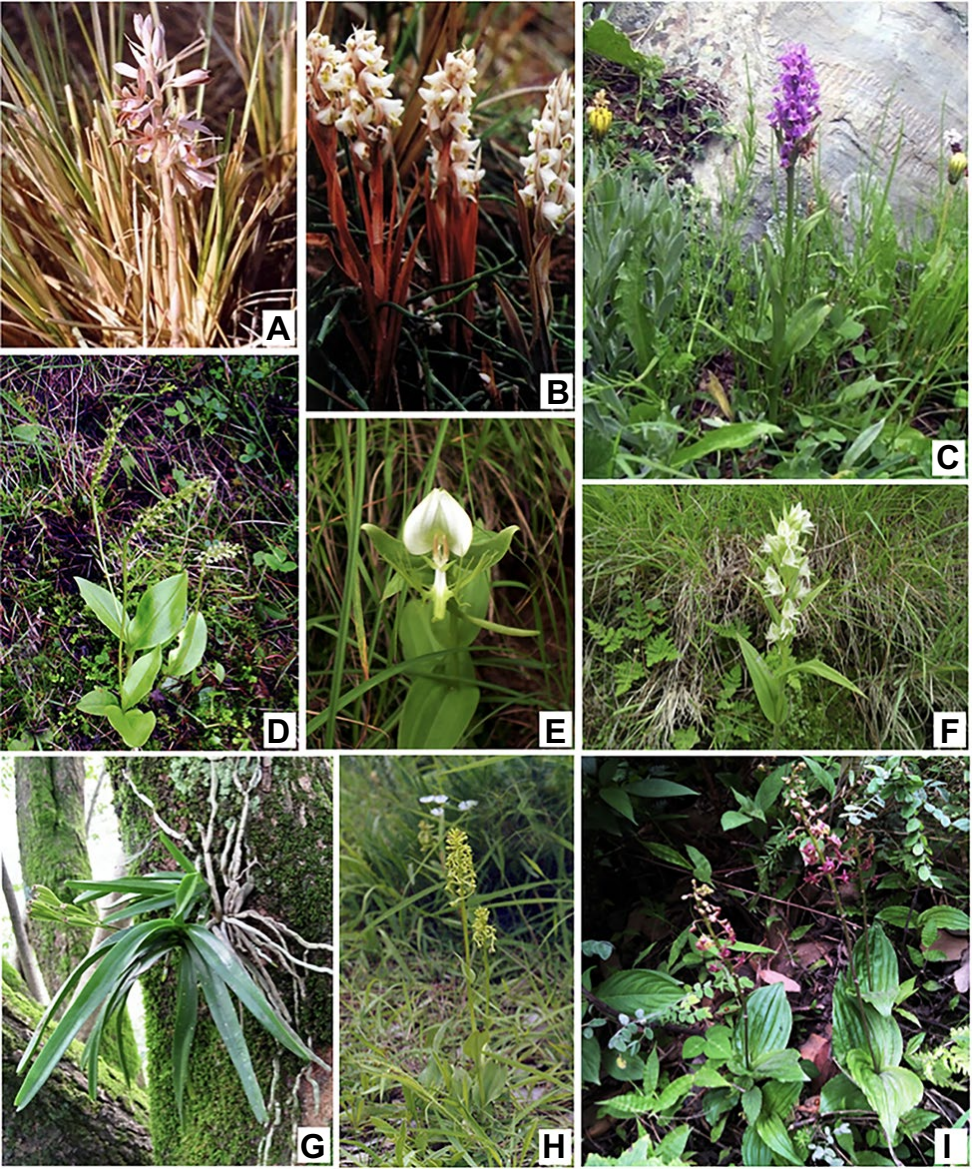
Some therapeutically important orchid species. **(A)**, *Eulophia dabia* (D.Don) Hochr.; **(B)**, *Zeuxine strateumatica* (L.) Schltr.; **(C)**, *Dactylorhiza hatagirea* (D.Don) Soó; **(D)**, *Malaxis muscifera* (Lindl.) Kuntze; **(E)**, *Habenaria intermedia* D.Don; **(F)**, *Habenaria pectinata* D.Don; **(G)**, *Vanda testacea* (Lindl.) Rchb.f.; **(H)**, *Platanthera edgeworthii* (Hook.f. ex Collett) R.K.Gupta; **(I)**, *Crepidium acuminatum* (D.Don) Szlach.

Orchids are members of one of the most advanced plant families, the Orchidaceae with their unique morphology (labellum, gynostemium), functional characteristics, and ecological adaptations (mycorrhizal association, and velamen) that are not found in model plants. Though popular as affluent ornamentals, orchids were first discovered for their therapeutic properties. The restorative properties of orchids have been well documented since times immemorial, Theophrastus in his book named “*Enquiry into Plants*” reported the use of orchids as therapeutics. These plants have also found reference in Indian and Chinese traditional pharmacopeia. In Indian Ayurvedic system of medicine, “*Ashtavarga*” is an important formulation, consisting of eight herbs, out of which four are orchids, that is, *Habenaria edgeworthii* (vriddhi), *Habenaria intermedia* (riddhi), *Malaxis acuminata* (jeevaka), and *Malaxis muscifera* (rishibhak). Similarly, in Chinese medicine, *Anoectochilus roxburghii* has been promoted as “*King medicine*” to treat snake bites, lung and liver disease, and hypertension (He et al., 2006). “*Shi-Hu*,” an orchid-based therapeutic formulation, prepared from *Dendrobium nobile* and allied species, is prized as a tonic because of its efficiency in treating lung, kidney, and stomach diseases, hyperglycemia, and diabetes (Bulpitt et al., 2007). “*Tian-Ma*” derived from tubers of *Gastrodia elata* is effectively used in the treatment of headaches, migraines, epilepsy, high blood pressure, rheumatism, fever, and nervous problems (Kong et al., 2003). In addition to their use as therapeutics, these plants have also been widely used as tonics and restoratives. The most important example is *Dactylorhiza hatagirea* which is used as an aphrodisiac (Lawler, 1984). Several orchids, such as Shwethuli (*Zeuxine strateumatica*) and Salabmisri (*Eulophia dabia*), *Vanda testacea*, and *Rhynchostylis retusa*, are used as aphrodisiacs, blood purifiers, general restorative tonics, and for treating rheumatism, piles, bronchitis, and inflammations (Chauhan, 1990; Vij et al., 2013; Hossain et al., 2020; Figure 1). These healing and restorative properties are due to the presence of a rich diversity of phytochemicals which are bioactive and are responsible for the pharmacognostic potential of these plants (Teoh, 2016).

The integration of traditional knowledge with modern research can pave a way as promising leads for the discovery of novel drugs with greater therapeutic potential than synthetic medicine offering new horizons in the field of therapeutics and drug discovery. However, the studies in this direction are not commensurate with the immense potential of these plants. This is mainly due to lack of complete understanding of the spectrum of molecular networks of secondary metabolism. Even though there have been a number of studies on the phytochemical profiling and the biological activity, there is limited information about the regulating molecular cues and the alternate biosynthetic routes which are utilized in these plants as a survival strategy in harsh and dynamic climatic conditions. Various omics approaches can be instrumental to understand and elucidate these complex mazes and help in utilization of these plants as therapeutics to their fullest potential.

Recent times have revolutionized the process of deciphering the genetic identity of the germplasm by using minimal amount of tissues to generate humongous volume of data using transcriptomic approach. Genome editing with the help of transcriptomic sequencing provide extra choices for genetic improvement in orchids. For techniques like CRISPR/Cas9, the sequence of the genome of the host can ascertain the specific and accurate target sites to increase the efficiency of the genome editing process (Kui et al., 2017) and can be highly beneficial for overall improvement of the germplasm. Transcriptomic sequencing has also helped in increasing the pace for the development of Simple Sequence Repeats (SSR), which are the microsatellite markers with random tandem repeats of 2–6 nucleotides. These markers are widely used because of their reproducibility, co-dominant nature, extreme polymorphism, simplicity, abundance, and easy amplification. The development of SSR markers in these medicinally important orchids can help in germplasm breeding, marker-assisted selection, parentage analysis, and genetic diversity studies. The SSR markers identified can help in evaluating and understanding genetic relationships quantitatively and qualitatively (Li et al., 2014) and help in constructing genetic maps of these plants which will further help in taxonomy, genetics, and genomic studies.

The reference genome of many medicinal non-model plants is not available. Transcriptomic approach provides an alternative way for collecting high-throughput data for gene identification, expression analysis, and putative functional characterization using metabolic profiling data (Góngora-Castillo and Buell, 2013). Whole transcriptome shotgun sequencing (WTSS) makes it possible to probe the genes of various metabolite biosynthesis processes and the relationship between the genes and plant metabolites. Another approach, termed as the Phytochemical genomics approach, involves sequence data sets combined with metabolomic data sets to elucidate the complete profile of secondary metabolites. In Digital gene expression analysis, differential expression of genes which are involved in secondary metabolism is studied to decipher the genetic variability and help in the drug discovery. The development of single-cell transcriptomics will aid in identifying networks and pathways and further facilitate drug discovery and development. The present study is an exhaustive review of the omics research on secondary metabolism in orchids, primarily focusing on the use of transcriptomic data for the analysis of genes and pathways associated with the synthesis of secondary metabolites and could be further be used for establishing the therapeutic potential of the orchids.

## Establishment of Orchids as Therapeutic Agents

The therapeutic potential of orchids has been reported since times immemorial. In 1579, Langham (1579) reported the antipyretic and anti-diarrheal properties of orchids. A Caribbean folklore mentions the use of *Vanilla claviculata* for treating wounds and syphilis (Griffith, 1847) while the flowers of *Vanilla griffithii* (Burkill, 1935) and leaf paste of *Vanda roxburghii* were used in treating fever (Chawla et al., 1992). *Dendrobium huoshanense* stems are reported to be beneficial for the eye, stomach, and liver ailments (Hsieh et al., 2008; Luo et al., 2008) while those of *Dendrobium monoliforme* are reported to be antipyretic (Zhao et al., 2003). Oil-based extracts of stems and leaves of *Anoectochilus formosanus* are effective for the treatment of hypertension, impotency, liver spleen disorders, and chest and abdominal pains (Satish et al., 2003). Leaf decoction of *Dendrobium candidum* is used for treating diabetes (Wu et al., 2004). Traditional usage of orchids as restoratives and tonics have been widely and commonly reported. The tubers of *Dactylorhiza hatagirea* have been used for the preparation of “*Salep*” which possess healing qualities (Lawler, 1984). Similar preparations like “*dbang lag*” have been used to provide sustenance for Tibetan monks practicing in remote caves (Teoh, 2019). Such studies coupled with ethnobotanical knowledge formed basis of many systematic reviews on utilization of orchids as therapeutic agents (Lawler, 1984).

Due to the significant role of orchids in the traditional medicine system, it has become imperative that these traditional remedies should be utilized for the discovery of new therapeutics. A plethora of studies has been reported ever since, to investigate the role of orchids as promising source of bioactive agents. A number of reports on the antioxidant and anti-inflammatory potential of various orchids like *Phalaenopsis* hybrids (Minh et al., 2016) and *Dendrobium officinale* (Zhang et al., 2017) have come up. Cytotoxic and apoptotic effects have also been reported in *Dendrobium crepidatum* and *D. chrysanthum* (Prasad and Koch, 2016). Antimicrobial activity has also been documented in *Dendrobium monoliforme* (Paudel et al., 2018). Antihyperglycemic (*Dactylorhiza hatagirea*; Choukarya et al., 2019), anti-diabetic and hepatoprotective activity (*Calanthe fimbriata*; Peng et al., 2019) have been reported.

To provide a sound scientific scaffolding for development of potential therapeutic products, efforts have been also directed to isolate and profile the phytochemicals from plant extracts. Various classes of secondary metabolites have been isolated from different plant parts and evaluated for biological activity. Phenanthrenes, like denbinobin, from *Dendrobium nobile*, showed potential cytotoxic activity (Lee et al., 1995), prevented metastatic gastric cancer, and showed potent therapeutic activity against hepatic fibrosis (Yang et al., 2007; Song et al., 2012). Similarly, kinsenoside from *Anoectochilus roxburghii* showed antihyperglycemic activity (Zhang et al., 2007). Cymbidine A from *Cymbidium goeringii* is responsible for the hypotensive and diuretic activity (Watanabe et al., 2007). Flavones C-glycosides and anthocyanins from red *Phalaenopsis* hybrids exhibited antioxidant activity (Kuo et al., 2010). Polysaccharides from *Dendrobium officinale* (Liu et al., 2011) and *Gastrodia elata* (Bao et al., 2017) have exhibited immune-enhancing potential. Galactoxyloglucan (GXG), a purified polysaccharide from *Dendrobium huoshanense*, improved insulin sensitivity, thus preventing hyperglycemia (Wang et al., 2019). Role of flavonoids especially rutin, in imparting antioxidant potential have also been highlighted in *Dendrobium officinale* (Zhang et al., 2017). Flavonoids of *Dactylorhiza hatagirea* also exhibited antihyperglycemic activity (Choukarya et al., 2019). Sesquiterpenoids from *Dendrobium nobile* exhibited neuroprotective activity (Ma et al., 2019b), while bibenzyl compounds from *Dendrobium officinale* showed cytotoxic activity (Ren et al., 2020). A group of compounds (phenanthrenes, bibenzyls, glucosyloxybenzyl succinate derivatives, flavonoids, lignans, terpenoids, etc.) isolated from *Pleione,* showed anti-tumor, anti-neurodegenerative, and anti-inflammatory biological activities (Wu et al., 2019). Despite a large number of reports on the phytochemical profiling in orchids, the studies are not commensurate with the immense potential of orchids as therapeutic agents. Omics techniques offer a great opportunity to provide an alternate and efficient method to study and characterize specific phytochemicals. Transcriptomic approaches can generate insights to the secondary metabolite biosynthetic pathways and can aid in functional characterization of their key regulatory genes.

## Transcriptomic DataSets in Orchids

Undeterred by the peculiarity in their unique characteristics, orchids are depreciated with respect to understanding their molecular complexities. A complete understanding of the spectrum of the molecular networks by isolated analyses of gene families is not plausible due to the limited availability of orchid genomes. On the other hand, transcriptome-wide analyses can help resolve complex metabolic pathways which are at play in these plants. Transcriptome is a complete set of mRNA and non-coding RNA produced by a cell or organism at a particular point of time. It generates large-scale transcripts that could help in analyzing different gene families all at once and could also guide toward understanding cross-links in mechanisms involved. The analysis begins with the collection of the desired tissue and subsequent isolation of RNA from the collected sample. The isolated RNA is used for the synthesis of complementary DNA which is eventually utilized for the construction of libraries after sequencing. There are large numbers of sequencing techniques that are prevalent nowadays, such as Roche/454, Illumina, Applied Biosystems SOLiD, and Helicos HeliScope (Magi et al., 2010). Even though these techniques produce abundant short reads at a much higher throughput than any Sanger sequencer but data presented after such analysis is a set of short reads composed of several hundred base pairs. The reads, thus, obtained are curated as raw reads. These read are first filtered and adjusted based on the quality control measures. Then the filtered reads are first either reconstructed using *de novo* assembly in absence of reference genome or assembled by alignment to the reference genome (Wolf, 2013). The assembly of the reads can be performed with tools like Trinity (Grabherr et al., 2011), Velvet (Zerbino and Birney, 2008), SPAdes (Bankevich et al., 2012), or SOAPdenovo-Trans (Xie et al., 2014). The assembled reads form contigs or singletons; both of these are part of unigenes. The functional annotation of the unigenes or transcripts is completed using various databases, such as NCBI,^1^ KEGG,^2^ and SwissProt.^3^ Additionally, the number of reads for a transcript provides the level of its abundance, thus serving as the starting point for biological inference of spatiotemporal gene expression (Wolf, 2013; Ma et al., 2019a). Transcriptome helps in identification of transcripts involved in primary and secondary metabolism and their splice variants (Wang et al., 2009). Comparing the levels of differentially expressed genes at different developmental stages or environmental conditions, provide insights into the physiological status of the tissue at a specific time. These data sets also contain information of small RNAs, long non-coding RNAs, and molecular repeats etc., and provide a tentative framework for functional assertion for putative annotations. These data can serve as an important lead for modern pharmaceutical industry toward development of herbal-based medicines.

High-throughput transcriptomic approaches produce extensive data sets that can be applied to identify candidate key genes in specific physiological processes using co-expression networks analysis (Carrera et al., 2009; Windram et al., 2014). On the other hand, targeted sequencing using degenerate primers proves to be economical and enables exhaustive analysis of specific genes. Specific genes exhibiting significant sequence similarity with gens involved in similar biological processes can be amplified by degenerate primers in related organisms (Wei et al., 2003). Functional validation of putative genes using metabolic profiling of flavonoids using gene-insertion mutants and transgenic plants with overexpressing genes could be used to understand the role genes in secondary metabolism. Further, recombinant proteins and *in vitro* biochemical assays could be used to decipher catalytic activity of the proteins. This “reverse genetics” approach for gene identification is very promising where bioinformatic prediction of candidate genes preceded the experimental analysis.

There have been a limited number of transcriptome-wide studies in orchids to explore and elucidate different aspects of orchid development (Table 1), however, the efforts are not in line with the immense advantage of using transcriptomic techniques to decipher various molecular networks. The therapeutic potential of orchids is closely associated with the intricate maze of secondary metabolism pathways and their by-products is mainly responsible for their diverse therapeutic properties. These pathways are, in turn, under strict control of an array of molecular factors which regulate the synthesis of phytochemicals. A large number of gene families are specifically associated with various biosynthetic pathways. Transcriptomic data emerging from various studies conducted in orchids have been tabulated in Table 1 and it is evident that Illumina sequencing was the most commonly used sequencing method and Trinity was the most common assembler software used. A maximum number of final reads were obtained in *Dendrobium officinale* (81,284,898; Yuan et al., 2020) and highest number of unigenes were identified in *Dendrobium huoshanense* (499,190, Zhou et al., 2020). A huge variation was noticed in the total number of unigenes as reported in different plant parts using different techniques. In *Dendrobium officinale*, the range in the number of unigenes was observed from 2,99,107 (Shen et al., 2017) to 23,131 (Adejobi et al., 2021) as reported from various tissues. Similarly, in *Dendrobium catenatum*, 23,139 unigenes were reported from stem tissue (Lei et al., 2018) and the number drastically increased to 478,361 in *Dendrobium huoshanense* when roots and leaves were also included for analysis (Yuan et al., 2018). This can be attributed to specific gene expression in tissues at various stages of growth and development and environmental conditions. In *Phalaenopsis amabilis*, a comparative number of unigenes were reported, 37,723 and 34,020, from petals and labellum, respectively (Yang et al., 2014), indicating that a similar genetic profile can be seen in tissues at comparable physiological stages. In *Anoectochilus roxburghii*, 186,865 unigenes were reported from root, stem, and leaves (Chen et al., 2020). Interestingly, different techniques and platforms used for sequencing analysis can also play a role in this variation. Root, stem, and leaf tissues of *Dendrobium huoshanense* reported 4,99,190 unigenes when the Illumina HiSeq2000 platform was used (Zhou et al., 2020) while 4,78,361 unigenes were identified when Illumina Hiseq 2500 platform was used (Yuan et al., 2018). Hence, it can be concluded that a lot of variation is observed in the transcriptomic data, and hence, the analysis needs to be supported with substantial functional studies.

**Table 1:** Enumeration of transcriptomic data in orchids.

Plant name	Sequencing platform	Assembly	Plant part	Raw reads		Final reads		Total unigenes	References
*Anoectochilus roxburghii*	Illumina HiSeq X Ten	Trinity	Roots, Stems, and Leaves	–		–		186,865	Chen et al., 2020
	Illumina HiSeq 2000	Trinity v.2.0.6 software	Non-mycorrhizal plant (NM)	NM	61,226,728	NM	61,071,914	–	Zhang et al., 2020a
					60,542,772		60,425,910		
					67,559,786		67,410,292		
			Mycorrhizal plant (M)	M	55,632,192	M	55,492,010		
					65,007,376		64,859,884		
					67,125,158		66,965,132		
*Bletilla striata*	Illumina Hiseq4000	Trinity	Leaves, tubers and roots	–		–		42,974 genes	Ma et al., 2021
*Bletilla striata* *(Thunb.) Reichb.f.* varieties	Illumina HiSeq 2000 platform	Trinity	Pseudobulbs	270,734,628		–		291,021	Chen et al., 2021
*Calanthe tsoongiana*	Illumina HiSeq X Ten	Trinity	Four transitional stages from seed to seedling	592,645,857		577,527,375		73,528	Jiang et al., 2021a
*Cymbidium goeringii*	Illumina HiSeq™ 2000 platform	Trinity	Floral bud, Half-flowering, Full flowering stage	161,763,530		159,616,374		85,868	Ramya et al., 2019
*Cymbidium kanran*	Illumina HiSeq™ 2,500	Trinity	Buds and flowers	–		–		181,335 transcripts and 74,713 unigenes	Zhou et al., 2021
*Cymbidium longibracteatum*	Illumina HiSeq 2000 platform	Trinity	Yellow leaves (YL)	YL	39,557,830	YL	5,685,015,511	116,422	Jiang et al., 2018
			Green leaves (GL)	GL	38,536,724	GL	5,503,245,825		
*Cymbidium tortisepalum* var. *longibracteatum* cultivars	Illumina HiSeq 2000 platform	Trinity	Green Rhizome (GR)	–		GR	39,557,830	134,527	Jiang et al., 2021b
							27,672,832		
							33,858,264		
							29,254,152		
			Yellow Rhizome (YR)			YR	38,536,724		
							33,875,625		
							30,554,768		
							26,698,355		
*Dactylorhiza hatagirea*	Illumina GA IIx platform	SOAP denovo-Trans	Leaves (L)	L	22,009,740	L	15,917,274	37,371	Dhiman et al., 2019
			Shoots (S)	S	21,263,988	S	15,456,424		
			Tubers (T)	T	25,884,232	T	17,788,506		
*Dendrobium catenatum*	Illumina HiSeqTM 4000	–	Stems	–		–		23,139	Lei et al., 2018
*Dendrobium huoshanense*	Illumina HiSeq 2000 platform	Trinity	Roots, Stems, and Leaves	476,746,678		444,999,698		499,190	Zhou et al., 2020
	Illumina HiSeq 2500 platform	Trinity	Roots, Stems, and Leaves	736,904,076		716,634,006		478,361	Yuan et al., 2018
*Dendrobium* Nestor (*Dendrobium parishii × D. anosmum*)	Illumina HiSeq™ 4000 platform	Trinity	Flower bud stage (F)	F	50,047,108	F	47,538,849	161,228	Cui et al., 2021
			Half bloom stage (H)	H	48,759,280	H	47,538,849		
			Full bloom stage (B)	B	51,171,054	B	48,879,555		
*Dendrobium nobile*	Illumina HiSeq 4000 platform	Trinity	Stems	43,01,49,656		41,48,90,782		207,283	Li et al., 2017
*Dendrobium officinale*	HiSeqTM 2500 Illumina	–	Roots Control (CK)	CK	83, 206, 690	CK	81,284,898	23,131	Adejobi et al., 2021
			MeJa treated (MeJa)	MeJa	82,623,796	MeJa	81,047,188		
	Illumina	–	Leaves	–		–		–	Zhang et al., 2021b
	BGISEQ-500	Trinity	Protocorm like bodies and Leaves	–		–		157, 901	Wang et al., 2020
	Illumina HiSeq 4000 platform	–	Roots, Stems, and Leaves	771,499,974		747,574,430		24,927	Yuan et al., 2020
	Illumina HiSeq 4000	Trinity 2.4.0	Leaves	–		269,267,462		60,597	Chen et al., 2019
	Illumina HiSeq 2500 platform	Trinity	Roots (R)	R	54,469,054, 71,462,678	R	54,433,348, 71,35,890	299,107	Shen et al., 2017
			Stems (S)	S	50,076,260, 64,920,086	S	50,076,260, 64,826,004		
			Leaves (L)	L	73,647,052, 53,904,216	L	73,534,024, 53,862,708		
			Flowers (F)	F	38,776,952, 38,669,310	F	38,736,660, 38,602,508		
	454 GS FLX Titanium platform	–	Stems	553,084		518,223		36,407	Guo et al., 2013
	Illumina HiSeq 4,000	–	Flowers of two cultivars Wanhu No.5 and Wanhu No.6	–		–		25,484 genes	Li et al., 2021a
*Dendrobium sinense*	–	–	Leaves and Pseudobulbs	568,756,484		563,154,602		72,797	Zhang et al., 2021a
*Gastrodia elata* hybrid (*Gastrodia elata BI.f.elata* × *Gastrodia elata BI.f.pilifera*)	Illumina HiSeq™ 2000	Trinity	Tuber	20,611,556		20,237,474		34,323	Wang et al., 2020
*Gastrodia elata*	BGISEQ-500 platform	Trinity	tuber, stem and flowers	–		–		113,067	Shan et al., 2021
*Ophrys exaltata, O. sphegodes* and *O. garganica*	454 and Solexa, Sanger sequencing	–	–	–		–		121,917 transcript	Sedeek et al., 2013
*Paphiopedilum armeniacum*	Illumina HiSeq4000	Trinity v2.4.0	Capsules	–		–		183,737	Fang et al., 2020
*Paphiopedilum hirsutissimum*	Illumina HiSeq™ 2000	Trinity (version: v2.9.0)	Flowers	–		18,236,750–21,697,775		28,805–34,806	Li et al., 2021b
*Phalaenopsis amabilis* white cultivar (Baiyuzan) Purple cultivar (Baolonghuanghou)	Illumina HiSeq 2500 platform	Trinity	Petals of White (WP) cultivar	WP	50,282,202	WP	19,744,124	114,293	Meng et al., 2019
					47,998,340		28,758,568		
					53,788,240		36,877,122		
			Petals of Purple (PP) cultivar	PP	49,944,218	PP	49,091,862		
					50,589,170		49,558,168		
					41,232,748		40,475,996		
*Phalaenopsis amabilis*	Illumina HiSeq 2000 system	Trinity	Petals (P)	–		P	10, 734, 813	37, 723	Yang et al., 2014
			Labellum (L)			L	16, 224, 038	34,020	
Red *Phalaenopsis* Dtps. Jiuhbao Red Rose Yellow *Phalaenopsis* Dtps. Fuller’s Sunset	Illumina HiSeq™ 2000	Trinity software (version trinityrnaseq_r2012-03-17)	Red Flower bud (RB)	–		RB	8,889,080	51,771	Gao et al., 2016
			Yellow Flower bud (YB)			YB	10,734,813		
*Phalaenopsis* hybrid: Konggangjinli	Illumina HiSeq2000	Trinity	Leaf	118,996,000		79,434,350		21, 348 genes 31,708 isogenes	Xu et al., 2015
*Pleione limprichtii*	Illumina HiSeqTM 4,000	Trinity	Flower petals and Lips	–		–		80,525	Zhang et al., 2020b
*Vanda “*Tan Chay Yan”	MiSeq Desktop Sequencer (Illumina)	CLC Genomic Workbench software Version 6.0	Tepals	4,955,918		4,826,959		–	Mohd-Hairul et al., 2020
*Vanilla planifolia*	454/Illumina	Velvet and Oases	Pods, Leaves, Stems and Roots	–		1,678,293		301,459 contigs	Rao et al., 2014

## Functional Annotation of Secondary Metabolism Specific Genes

Transcriptomic data can provide a basic lead for functional studies if a unified, systematic, and statistically significant approach is adopted for its assembly and characterization. To scrutinize the functionality of the unigenes identified from the transcriptomic data set, their assessment was carried out against different databases like KEGG, Swissprot, and non-redundant database (Nr; Table 2). The highest similarity of the unigenes was found against the Nr database except in the case of *Dendrobium huoshanense* where the SwissProt similarity of unigenes was the highest (Zhou et al., 2020). Out of 186,865 unigenes identified in *Anoectochilus roxburghii,* approximately 35, 32, and 47% were annotated using KEGG, SwissProt, and Nr database (Chen et al., 2020). However, only 9,946 out of 73,528 unigenes were annotated by KEGG in *Calanthe tsoongiana* (Jiang et al., 2021a). In *Dendrobium officinale*, the unigenes characterized using SwissProt varied from 13,418 (Guo et al., 2013) to 62,695 unigenes (Wang et al., 2021). The variation could be due to the use of different platforms used for sequencing or assembly and due to the type of tissue used in different studies.

**Table 2:** Functional Annotation using KEGG, SwissProt, and non-redundant (Nr) database.

Plant name	KEGG	SwissProt	Nr database	References
*Anoectochilus roxburghii*	66,542 unigenes	59,736 unigenes	87,781 unigenes	Chen et al., 2020
*Calanthe tsoongiana*	9,946 unigenes	25,124 unigenes	35,368 unigenes	Jiang et al., 2021a
*Cymbidium goeringii*	33,417 unigenes	36,911 unigenes	54,640 unigenes	Ramya et al., 2019
*Cymbidium longibracteatum*	10,723 unigenes	21,297 unigenes	33,487 unigenes	Jiang et al., 2018
*Cymbidium tortisepalum* var. *longibracteatum*	44,141	44,577	70,576	Jiang et al., 2021b
*Dactylorhiza hatagirea*	9,130 transcripts	–	21,695 transcripts	Dhiman et al., 2019
*Dendrobium catenatum*	4,203 unigenes	–	–	Lei et al., 2018
*Dendrobium huoshanense*	112,603 unigenes	225,268 unigenes	140,919 unigenes	Zhou et al., 2020
	108,417 unigenes	101,132 unigenes	196,739 unigenes	Yuan et al., 2018
*Dendrobium nobile*	18,911 unigenes	48,431 unigenes	56,378 unigenes	Li et al., 2017
*Dendrobium officinale*	71,648 unigenes	62,695 unigenes	99,474 unigenes	Wang et al., 2021
	12,877 genes	18,804 genes	29,229 genes	Chen et al., 2019
	65,286 unigenes	38,765 unigenes	70,146 unigenes	Shen et al., 2017
	20,274 unigenes	13,418 unigenes	22,752 unigenes	Guo et al., 2013
*Gastrodia elata* hybrid (*Gastrodia elata BI.f.elata* × *Gastrodia elata BI.f.pilifera*)	8,364 unigenes	19,028 unigenes	24,230 unigenes	Wang et al., 2020
*Gastrodia elata*	56,585	52,164	71,069	Shan et al., 2021
*Ophrys exaltata, O. sphegodes* and *O. garganica*	7,394 transcripts	–	–	Sedeek et al., 2013
*Paphiopedilum armeniacum*	12,141 unigenes	44,893 unigenes	89,289 unigenes	Fang et al., 2020
*Phalaenopsis amabilis* white cultivar (Baiyuzan) and purple cultivar (Baolonghuanghou)	16,777 unigenes	–	48,071 unigenes	Meng et al., 2019
Red *Phalaenopsis* Yellow *Phalaenopsis*	5,446 unigenes	19,446 unigenes	27,084 unigenes	Gao et al., 2016
*Phalaenopsis* hybrid: Konggangjinli	14,099 unigenes	–	–	Xu et al., 2015
*Pleione limprichtii*	11,067 unigenes	21,177 unigenes	33,459 unigenes	Zhang et al., 2020b
*Vanilla planifolia*	–	–	130,550 unigenes	Rao et al., 2014

The annotation of genes or transcripts obtained using various servers helped in the characterization of genes based on their functional roles. The KEGG analysis of different studies in association with pathways of secondary metabolism has been summarized in Table 3. KEGG analysis of stem, leaves, and roots revealed the presence of cyanoamino acid metabolism, phenylpropanoid biosynthesis, diterpenoid biosynthesis, flavonoid and flavonol biosynthesis, steroid biosynthesis, and isoflavonoid biosynthesis pathways in *Anoectochilus roxburghii* (Chen et al., 2020). 65,286 unigenes in *Dendrobium officinale* (Shen et al., 2017), 10,723 unigenes in *Cymbidium longibracteatum* (Jiang et al., 2018), and 9,130 unigenes in *Dactylorhiza hatagirea* (Dhiman et al., 2019) were annotated by KEGG analysis. Differential gene expression (DEG) of the different colored buds of *Phalaenopsis* sp. suggested that most DEGs were of phenylpropanoid biosynthesis which suggests the role of anthocyanins for variable colors (Gao et al., 2016). The number of unigenes annotated to phenylpropanoid varies from 49 unigenes in *Phalaenopsis* sp. (Gao et al., 2016) to 466 in *Cymbidium goeringii* (Ramya et al., 2019). In a transcriptomic study of *Pleione limprichtii*, 11,067 genes were mapped to 131 KEGG pathways and 1,294 unigenes were associated with secondary metabolite synthesis (Zhang et al., 2020b).

**Table 3:** KEGG pathway analysis of secondary metabolism.

Plant name (*Reference*)	Unigenes/transcripts	Secondary metabolism		
		Pathway	Unigenes/transcripts	
*Anoectochilus roxburghii* (Chen et al., 2020)	66,542 unigenes	Biosynthesis of other secondary metabolites	Root	3,369 unigenes
			Stem	3,302 unigenes
			Leaf	3,280 unigenes
*Calanthe tsoongiana* (Jiang et al., 2021a)	9,946 unigenes in 25 pathways	Biosynthesis of other secondary metabolites	290 unigenes	
*Cymbidium goeringii* (Ramya et al., 2019)	33,417 unigenes	Anthocyanin biosynthesis	9 unigenes	
		Indole alkaloid biosynthesis	21 unigenes	
		Isoflavonoid biosynthesis	36 unigenes	
		Tropane, piperidine and pyridine alkaloid biosynthesis	50 unigenes	
		Isoquinoline alkaloid biosynthesis	51 unigenes	
		Monoterpenoid biosynthesis	56 unigenes	
		Sesquiterpenoid and triterpenoid biosynthesis	75 unigenes	
		Flavone and flavonol biosynthesis	134 unigenes	
		Diterpenoid biosynthesis	172 unigenes	
		Terpenoid backbone biosynthesis	197 unigenes	
		Flavonoid biosynthesis	236 unigenes	
		Phenylpropanoid biosynthesis	466 unigenes	
		Biosynthesis of secondary metabolites	3,197 unigenes	
*Dendrobium catenatum* (Lei et al., 2018)	4,203 unigenes	Flavonoid biosynthesis	31 unigenes	
*Dendrobium huoshanense* (Zhou et al., 2020) (Yuan et al., 2018)	112,603 unigenes annotated in 131 pathways	Biosynthesis of other secondary metabolites	2,237 unigenes	
	108,417 unigenes annotated to 33 pathways	Biosynthesis of other secondary metabolites	1,298 unigenes	
*Dendrobium nobile* (Li et al., 2017)	18,911 unigenes assigned to 131 pathways	Biosynthesis of other secondary metabolites	507 genes	
*Dendrobium officinale* (Chen et al., 2019)	12,877 genes grouped into 19 secondary level pathways	Biosynthesis of other secondary metabolites	716 genes	
*Dendrobium officinale* (Guo et al., 2013)	20,274 unigenes	Biosynthesis of other secondary metabolites	5 unigenes	
*Gastrodia elata* hybrid (*Gastrodia elata BI.f.elata* × *Gastrodia elata BI.f.pilifera*) (Wang et al., 2020)	8,364 unigenes	Phenylpropanoid biosynthesis	92 unigenes	
		Flavonoid biosynthesis	39 unigenes	
		Flavone and flavonol biosynthesis	18 unigenes	
		Tropane, piperidine and pyridine alkaloid biosynthesis	13 unigenes	
		Isoquinoline alkaloid biosynthesis	8 unigenes	
		Anthocyanin biosynthesis	1 unigene	
*Ophrys exaltata* *O. sphegodes* *O. garganica* (Sedeek et al., 2013)	7,394 transcripts	Biosynthesis of other secondary metabolites	252 transcripts	
*Phalaenopsis amabilis* white cultivar (Baiyuzan) *Phalaenopsis amabilis* purple cultivar (Baolonghuanghou) (Meng et al., 2019)	16,777 unigenes assigned to 129 pathways	Phenylpropanoid synthesis	168 genes	
		Flavonoid synthesis	39 genes	
		Flavone and flavonol synthesis	19 genes	
		Anthocyanin synthesis	7 genes	
		Biosynthesis of other secondary metabolites	328 genes	
Red *Phalaenopsis* Dtps. Jiuhbao Red Rose Yellow *Phalaenopsis* Dtps. Fuller’s Sunset (Gao et al., 2016)	5,446 unigenes	Phenylpropanoid biosynthesis	49 genes	
		Flavonoid biosynthesis	21 genes	
		Indole alkaloid biosynthesis	1 gene	
		Flavone and Flavonol biosynthesis	13 genes	
		Isoquinoline alkaloid biosynthesis	11 genes	
*Phalaenopsis* hybrid: Konggangjinli (Xu et al., 2015)	14,099 unigenes assigned to 123 pathways	Biosynthesis of secondary metabolites	791 unigenes	
		Terpenoid backbone biosynthesis	55 unigenes	
		Indole alkaloid biosynthesis	1 unigene	
		Monoterpenoid biosynthesis	1 unigene	
		Diterpenoid biosynthesis	20 unigenes	
		Sesquiterpenoid and triterpenoid biosynthesis	4 unigenes	
		Phenylpropanoid biosynthesis	75 unigenes	
		Flavonoid biosynthesis	34 unigenes	
		Flavone and flavonol biosynthesis	15 unigenes	
		Isoquinoline alkaloid biosynthesis	9 unigenes	
		Tropane, piperidine and pyridine alkaloid biosynthesis	20 unigenes	
*Pleione limprichtii* (Zhang et al, 2020b)	11,067 unigenes mapped onto 131 pathways	Biosynthesis of secondary metabolites	1,294 unigenes	
		Phenylpropanoid biosynthesis	167 unigenes	
		Terpenoid backbone biosynthesis	53 unigenes	
		Flavonoid biosynthesis	36 unigenes	
		Diterpenoid biosynthesis	35 unigenes	
		Isoquinoline alkaloid biosynthesis	28 unigenes	
		Tropane, piperidine and pyridine alkaloid biosynthesis	24 unigenes	
		Flavone and flavonol biosynthesis	7 unigenes	
		Sesquiterpenoid and triterpenoid biosynthesis	5 unigenes	
		Anthocyanin biosynthesis	1 unigene	

**Figure 2:**
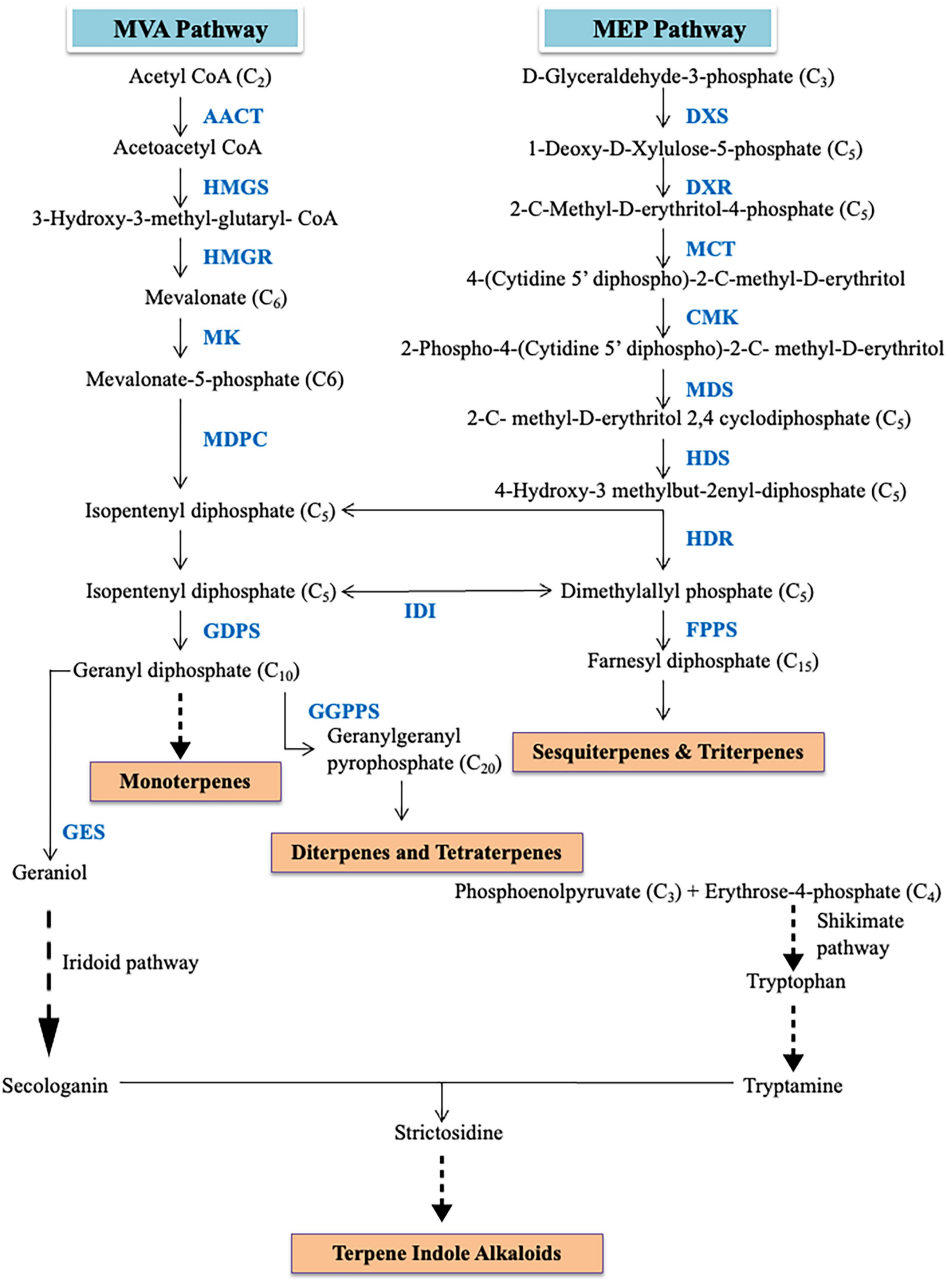
Diagrammatic representation of methyl-d-erythritol 4-phosphate (MEP) and mevalonate (MVA) pathway.

Even though large numbers of secondary metabolites are produced by plants, only a selected compounds have important medicinal properties. These secondary metabolites can be grouped into various classes like alkaloids, terpenoids, polyphenols, phenanthrene, bibenzyl derivatives, etc. Therapeutic effects of different alkaloids especially terpenoid alkaloids have been widely reported in orchids (Sut et al., 2017; Gantait et al., 2021; Ghai et al., 2021). These terpenes alkaloids are formed through the mevalonate (MVA) pathway and methyl-D-erythritol 4-phosphate (MEP) pathway (Figure 2). MVA pathway initiates with acetyl-CoA as a precursor. Acetyl-CoA undergoes a series of catalyzation reactions to produce isopentyl pyrophosphate (IPP) and dimethylallyl pyrophosphate (DMAPP). These IPP units are further processed to form sesquiterpenes. Meanwhile, the MEP pathway begins with the condensation of pyruvate and D-glyceraldehyde-3-phosphate by 1-Deoxy-D-xylulose-5-phosphate synthase (DXS). The regulatory mechanisms and biochemistry of the mevalonate (MVA) and methyl-D-erythritol 4-phosphate (MEP) pathway are well characterized. In MVA, 3-hydroxy-3-methylglutaryl coenzyme A reductase (HMGR) lays an important role in controlling the metabolic flux. The regulation of mevalonate kinase (MK) is regulated by feedback mechanism at both transcriptional and post-translational levels (Hinson et al., 1997). The isopentyl pyrophosphate (IPP) units are isomerized into dimethylallyl pyrophosphate (DMAPP) by IPP isomerase which is the initiating molecule in terpenoid biosynthesis. The enzyme, 1-deoxy-D-xylulose-5-phosphate synthase (DXS), is key player in controlling influx into the MEP pathway through decarboxylation reaction and is regulated by feedback mechanism through IPP and DMAPP (Banerjee et al., 2013). This can be corroborated by the higher expression of DXS and the terpenoid levels in the inflorescences in *Arabidopsis* (Carretero-Paulet et al., 2002). This process proceeds to form IPP and DMAPP *via* multistep reactions catalyzed by a series of enzymes. The MEP and MVA pathways are both linked by an intermediary precursor isopentenyl pyrophosphate. Subsequently, the pathways result in the formation of monoterpenoids, diterpenoids, carotenoids, sesquiterpenoids, and some other metabolites. Sesquiterpene alkaloids are the most abundant types of alkaloids of *Dendrobium* (Chen et al., 2019). Hsiao et al. (2011) reported the identification of 50 unigenes of the MEP and MVA pathways in *Phalaenopsis* while in *Cymbidium goeringii*, 32 unigenes of MVA and 38 unigenes of MEP pathway were identified (Ramya et al., 2019). Forty-six unigenes in *Dendrobium huoshanense* (Yuan et al., 2018) and 36 in *Dendrobium officinale* (Shen et al., 2017) related to the MEP and MVA pathway were identified. According to Li et al. (2017), isoprene units obtained through the MEP pathway were responsible for the biosynthesis of dendrobine in *Dendrobium nobile*. The expression of *acetyl-CoA acetyltransferase* (*AACT*), *mevalonate diphosphosphate decarboxylase* (*MVD*), *phosphomevalonate kinase* (*PMK*), and *Alpha-humulene synthase* (*TPS21*) changes upon inoculation of the orchid with MF23 (*Mycena* sp.) which results in induction of pathway leading to dendrobine biosynthesis (Li et al., 2017). Besides fungal stimulation, methyl jasmonate (MeJA) treatment of *D. officinale* also results in increased expression of genes associated with MEP and MVA pathway (Chen et al., 2019). Toh et al. (2017) also studied the fragrant sites in *Vanda* Mimi Palmer which indirectly points toward the sites of high monoterpenoid production. Higher expression of *3-hydroxy-3-methylglutaryl coenzyme A reductase* (*HMGR*) and *1-deoxyxylulose-5-phosphate synthetase* (*DXS*) was observed in root than in leaf but *DXS* and *1-deoxy-D-xylulose-5-phosphate reductoisomerase* (*DXR*) were abundant mainly in stems of *Dendrobium huoshanense* (Yuan et al., 2018). Yuan et al. (2018) suggested stem-specific accumulation of alkaloids in *D. huoshanense* but leaf-specific accumulation is observed in *D. officinale* (Shen et al., 2017). A series of enzymes associated with strictosidine were also identified in a study on *Dendrobium officinale* (Shen et al., 2017). Accumulation of dendrobine, a sesquiterpene alkaloid, was consistently more when the expression of *PMK* and *MVD* was high but got reduced as the expression of the aforementioned genes decreased in MF23 infected *Dendrobium nobile* orchid plant (Li et al., 2017). In the same study, the dendrobine pathway was negatively correlated with the expression of *TPS21* but no relation with genes of MEP was observed (Li et al., 2017). Secologanin synthase (SCS) which is essential for the synthesis of secologanin has also been reported to be involved in alkaloid biosynthesis (Guo et al., 2013). Different terpenes are synthesized from isopentenyl diphosphate through two pathways mevalonate pathway and methylerythritol phosphate pathway. Hsiao et al. (2006) analyzed transcriptomes of *Phalaenopsis bellina* and *Phalaenopsis equestris* where genes related to the DXP-geraniol linalool pathway were identified by data mining. In another study, regulation of monoterpene biosynthesis by PbbHLH4 in *Phalaenopsis* orchid was provided (Chuang et al., 2018). Terpene synthases (TPSs) are responsible for the structure diversity of terpene while cytochromes P450 (CYPs) further modifies the products from TPSs which provide further diversification of terpenes (Tsai et al., 2017). In *D. huoshanense*, 229 unigenes of the P450 superfamily were identified (Yuan et al., 2018) but in *D. officinale*, 236 unigenes associated with P450 were mined (Shen et al., 2017). Strictosidine synthase had higher expression levels in protocorm like bodies (PLBs) than in leaves suggesting the higher content of total alkaloid is related to the higher amount of precursor strictosidine produced in *D. officinale* (Wang et al., 2021). The positive stimulation of either MEP or MVA pathway could eventually lead to an increase in the production of alkaloids which could eventually increase the therapeutic potential of the orchid plant.

**Figure 3:**
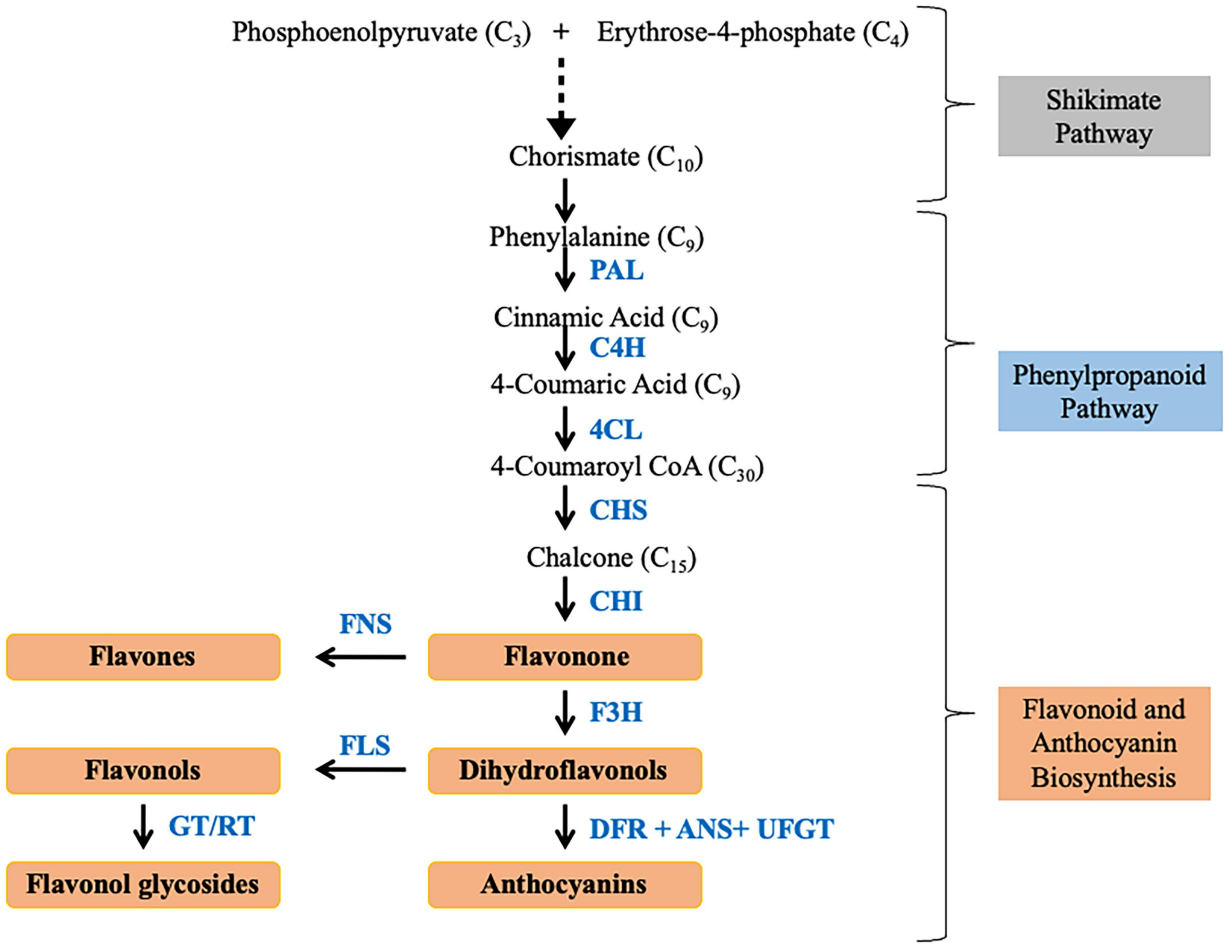
Schematic flowchart depicting flavonoid and anthocyanin biosynthesis.

Besides alkaloids, the role of flavonoids as antioxidant, anti-cancer, and anti-aging agents has also been highlighted (Middleton et al., 2000). The flavonoids are compounds with bridged phenyl rings which are synthesized through the phenylpropanoid pathway. Flavonoid also provides resistance against disease and insects in plants and enable the plant for adapting to adverse environmental conditions with the help of increased production in secondary metabolites (Campos and Hamdan, 2000; Yuan et al., 2020). *Anoectochilus roxburghii* is rich in flavonoid compounds, such as dihydroquercetin, quercetin, kaempferol, and myricetin (Ye et al., 2017), which are responsible for the drug activity of this orchid plant (Chen et al., 2020). Lei et al. (2018) reported about C-glycosides type flavonoids are more abundant than O-glycosides in *Dendrobium*. The metabolic analysis of *Anoectochilus roxburghii* revealed an abundance of flavonoids in leaves than in roots or stems (Chen et al., 2020). The by-products of the shikimate pathway are the precursor for a large assortment of secondary metabolites (Tzin et al., 2012; Takayuki et al., 2013). It is a multistep process that starts with the condensation of phosphoenolpyruvate (PEP) and erythrose 4-phosphate (E4P; Figure 3). The intermediate chorismite after further processing leads to the independent formation of aromatic amino acids, tryptophan, tyrosine, phenylalanine. Phenylalanine is the precursor for the Phenylpropanoid pathway which ultimately results in the synthesis of flavonoids. PAL is the most important rate limiting fulcrum enzyme that links primary metabolism with secondary metabolism (Vogt, 2010; Fraser and Chapple, 2011). A positive correlation between the PAL enzyme activity and accumulation of phenylpropanoid compounds has been widely reported (Bate et al., 1994; Vogt, 2010). Carbon flux into different branches of flavonoid synthesis is regulated by flavonol synthase (FLS; Davies et al., 2003). In *Arabidopsis*, activity of hydroxycinnamoyl-CoA shikimate/quinate hydroxycinnamoyl transferase (HCT) led to maneuvering of the metabolic flux into flavonoids through Chalcone synthase (CHS) activity (Besseau et al., 2007). Additionally, there are several transcription factors that regulate the gene expression which ultimately controls the metabolic flux. The expression of regulatory molecules like MYB is inversely proportional to lignin production, thus facilitating the metabolic flux toward flavonoid production (Fornale et al., 2010). Similarly, elicitors like salicylic acid and methyl jasmonate positively diverts the metabolic flux toward increased production of secondary metabolites (Creelman and Mullet, 1997; Kessler and Baldwin, 2002). Phenylalanine is catalyzed by phenylalanine ammonia lyase (PAL) to form cinnamate which is converted to *p*-coumaroyl-CoA by trans-cinnamate 4-monooxygenase (C4H) and 4-coumaroyl-CoA synthase (4CL). *p*-coumaroyl-CoA is further processed by series of different enzymes to form flavonoids, flavonols, flavanones, and anthocyanins.

A total of 15 unigenes encoding seven enzymes of the flavonoid pathway were identified from *D. huoshanense* (Zhou et al., 2020) while 31 and 19 unigenes in *D. catenatum* (Lei et al., 2018) and *Pleione limprichtii* (Zhang et al., 2020b). In a study on *Anoectochilus roxburghii*, inoculation with *Ceratobasidium* sp. AR2 increases the flavonoid content of the plant by upregulating *PAL*, *chalcone synthase* (*CHS*), *4CL* and downregulating of *cinnamate 4-hydroxylase* (*C4H*), and *chalcone isomerase* (*CHI*) genes (Zhang et al., 2020a). In a new cultivar of *Cymbidium longibracteatum* with yellow leaves and tubers, seven unigenes related to flavonoid biosynthesis were upregulated (Jiang et al., 2018). Similarly, expression levels of *CHS*, *CHI*, *dihydroflavonol 4-reductase* (*DFR*), *anthocyanidin synthase* (*ANS1*), and *UDP-glucose: flavonoid-3-O-glucosyltransferase* (*UFGT*) were comparatively higher in red, corroborating with higher anthocyanin content in the red stems of *D. candidum* (Jia et al., 2021). Similarly, most of the genes involved in anthocyanin biosynthesis were upregulated during floral development of *Dendrobium* nestor (Cui et al., 2021). Expression of *PAL* and *HMG-CoA* reductase was upregulated in the abaxial surface of the tissue of *Vanda* Mimi Palmer (Toh et al., 2017). The rate of flavonoid production in plants was reported to be controlled by *CHS* with associated *CHI*. The higher expression levels of *CHS*, *CHI*, *flavonol synthase* (*FLS*), *DFR*, and *Anthocyanidin reductase* (*ANR*) in roots than in stems and leaves of *A. roxburghii* were reported as well (Chen et al., 2020). Upregulation of *LAR1*, *DFR3*, *flavanone 3-hydroxylase* (*F3H*), *CHS1*, *CHS2*, and *CHS3* in leaves facilitates the copious accumulation of flavonoids in leaves of *Dedrobium officinale* (Yuan et al., 2020). In the same study, *Dihydroflavonol reductase* (*DFR*), which is responsible for the conversion of flavonoids into anthocyanin biosynthesis, has higher expression in stems and leaves. MeJA treatment in *D. officinale* lead to the accumulation of bibenzyl (erianin and gigantol) increased due to upregulation of *PAL*, *4CL*, *C4H*, and *CYP450* (Adejobi et al., 2021). During explant browning in *Phalaenopsis* sp., higher expression of *PhPAL*, *PhCHS*, and *Ph4CL* was observed which suggest the role of anthocyanin in the early stages of tissue browning (Xu et al., 2015). Similarly, upregulated expression of *Pa4CL*, *PaANS*, *PaF3H*, and *PaDFR* was detected in purple petal cultivar of *Phalaenopsis amabilis* (Meng et al., 2019). The study on *Phalaenopsis* did not identify any DEGs related to *CHS*, *ANS*, *DFR*, and *flavonoid-3′-hydroxylase* (*F3*′*H*) in white petals which could be due to either technical limitations or due to absence of anthocyanin pathway (Yang et al., 2014). Similarly, no transcript of flavonoid-3′,5′-hydroxylase (*F3*′*5*′*H*) was identified from the transcriptome of *Ophrys* even though 61 transcripts of anthocyanins pathway were mined (Sedeek et al., 2013). Expression of *PlCHS*, *PlCHI*, and *PlFLS* was upregulated in white petals but colored petals had higher expression of *PlF3’H*, *PlDFR,* and *PlANS* in *Pleione limprichtii* (Zhang et al., 2020b). *PAL*, *4CL*, and *C4H* were upregulated in 8 and 10 weeks old seeds of *Vanilla planifolia* (Rao et al., 2014). Expression of *trans-resveratrol-di-O-methyltransferase-like* (*ROMT*) encoding gene, responsible for resveratrol biosynthesis, was high in tubers of *Dactylorhiza hatagirea* (Dhiman et al., 2019). It positively correlates with the fact that tubers of this plant are used as anti-inflammatory, anticarcinogenic, and as a cardioprotective agent. Higher expression of *ROMT* correlated with the abundant quantity of resveratrol and stilbenes (Dhiman et al., 2019). The role of caffeic acid, coumaric acid, and Caffeoyl CoA in the synthesis of resveratrol and stilbenes has also been pointed out in the same study. Genes associated with flavonoid pathways were reported to be regulated by UDP-glycosyltransferase and cytochrome P450 (Liu et al., 2013). DcTT8, a bHLH transcription factor in *D. candidum*, regulated the anthocyanin production by binding to the promoter region of *DcF3′H* and *DcUFGT* (Jia et al., 2021). The above review asserts that transcriptomic approaches can serve as a boon for gene discovery, functional annotation, and expression profiling in non-model organisms.

## Conclusion

Orchids grow in a variety of habits and habitats mainly owing to the presence of an array of unique secondary metabolites which help these plants sustain the stressful conditions. Therefore, these plants have emerged as important source for bioprospecting following traditional approaches. Omics technology, on the other hand, offer great potential for analysis of the complete metabolic pathways and provides detailed insights to gene function for drug discovery and other therapeutic interventions. The present study is a comprehensive analysis of transcriptomes more than 30 orchids mainly focusing on the alkaloids and flavonoids pathways. It can form the basis of an effective resource for the functional studies on tapping the immense potential of unique orchid secondary metabolites to facilitate development of novel therapeutic products from these plants.

## Author Contributions

JS conceptualized the work. DG and AK performed the analysis and prepared the original draft. PK, SP, and JS critically reviewed and edited the draft. All the authors have read and approved the final version.

## Conflict of Interest

The authors declare that the research was conducted in the absence of any commercial or financial relationships that could be construed as a potential conflict of interest.

## Publisher’s Note

All claims expressed in this article are solely those of the authors and do not necessarily represent those of their affiliated organizations, or those of the publisher, the editors and the reviewers. Any product that may be evaluated in this article, or claim that may be made by its manufacturer, is not guaranteed or endorsed by the publisher.

## Footnotes

^1^ https://www.ncbi.nlm.nih.gov/

^2^ https://www.genome.jp/kegg/

^3^ https://www.expasy.org/resources/uniprotkb-swiss-prot
